# Platelet in thrombo-inflammation: Unraveling new therapeutic targets

**DOI:** 10.3389/fimmu.2022.1039843

**Published:** 2022-11-14

**Authors:** Swati Sharma, Tarun Tyagi, Silvio Antoniak

**Affiliations:** ^1^ UNC Blood Research Center, School of Medicine, University of North Carolina at Chapel Hill, Chapel Hill, NC, United States; ^2^ Yale Cardiovascular Research Center, Yale School of Medicine, New Haven, CT, United States

**Keywords:** platelets, hemostasis, thrombo-inflammation, signaling, infections

## Abstract

In the broad range of human diseases, thrombo-inflammation appears as a clinical manifestation. Clinically, it is well characterized in context of superficial thrombophlebitis that is recognized as thrombosis and inflammation of superficial veins. However, it is more hazardous when developed in the microvasculature of injured/inflamed/infected tissues and organs. Several diseases like sepsis and ischemia-reperfusion can cause formation of microvascular thrombosis subsequently leading to thrombo-inflammation. Thrombo-inflammation can also occur in cases of antiphospholipid syndrome, preeclampsia, sickle cell disease, bacterial and viral infection. One of the major contributors to thrombo-inflammation is the loss of normal anti-thrombotic and anti-inflammatory potential of the endothelial cells of vasculature. This manifest itself in the form of dysregulation of the coagulation pathway and complement system, pathologic platelet activation, and increased recruitment of leukocyte within the microvasculature. The role of platelets in hemostasis and formation of thrombi under pathologic and non-pathologic conditions is well established. Platelets are anucleate cells known for their essential role in primary hemostasis and the coagulation pathway. In recent years, studies provide strong evidence for the critical involvement of platelets in inflammatory processes like acute ischemic stroke, and viral infections like Coronavirus disease 2019 (COVID-19). This has encouraged the researchers to investigate the contribution of platelets in the pathology of various thrombo-inflammatory diseases. The inhibition of platelet surface receptors or their intracellular signaling which mediate initial platelet activation and adhesion might prove to be suitable targets in thrombo-inflammatory disorders. Thus, the present review summarizes the concept and mechanism of platelet signaling and briefly discuss their role in sterile and non-sterile thrombo-inflammation, with the emphasis on role of platelets in COVID-19 induced thrombo-inflammation. The aim of this review is to summarize the recent developments in deciphering the role of the platelets in thrombo-inflammation and discuss their potential as pharmaceutical targets.

## 1 Introduction

Platelets originate from megakaryocytes as anucleate cells equipped with their own survival machinery to function in circulation. The main hemostatic role of platelets is to form the platelet plug at the site of vascular lesion or injury, to limit the blood loss and thus maintain hemostasis. Importantly, under pathological conditions, the dysregulated intravascular activation and aggregation of platelets along with activation of local coagulation factors lead to formation of vessel-occluding thrombi. This series of events may result in ischemia and infarction of affected tissues. Increasing number of reports revealing the complexity associated with platelet signaling pathways have encouraged researchers to dig deeper into exploring the role of platelets beyond hemostasis. Prior to last few decades, platelets were not studied for their role in the pathogenesis of infectious diseases. Their key role was thought to be limited as primary effectors of hemostasis and thrombosis, with very few studies exploring platelets in host immune response during various infectious diseases.

In the past two decades, platelets have been extensively explored for their involvement in different physiological processes including inflammation, vascular permeability, and tissue repair (1–5). There is a growing number of evidence from literature that emphasize on the role of platelets as main sentinel and effector cells involved in bridging hemostasis, inflammation, and the immune system. The role of platelets now extends beyond their traditional role as marginal mediators of hemostasis and thrombosis, as the contributors to diverse immunological processes. The recent investigations provide substantial evidence for their role against microbial threats, modulating antigen presentation, enhancement of adaptive immune responses, recruitment and promotion of innate effector cell functions. Platelets until last decade were underappreciated orchestrator of the immune system but now emerged as crucial players during infectious diseases too, as recent studies document the role of platelets in inflammation, hemostasis, and maintenance of vascular integrity during infection (3, 6–10). As platelets through different receptors can respond to both PAMPs (Pathogen associated molecular patterns) and DAMPs (Damage associated molecular patterns) released during non-sterile (infection) and sterile inflammation, it becomes pertinent to investigate their role and mechanism of action in both kind of inflammation. Investigating the role of platelets in both sterile and non-sterile inflammation will not only shed the light on shared molecular mechanism but can also reveal novel therapeutic targets.

The platelets are highly sensitive to environmental cues. These environmental stimuli tightly regulate not only hemostatic and immunomodulatory functions of platelets but also their interaction with the vasculature (endothelium) and components of innate immunity. Platelets can sense intravascular invasion of pathogen, whether its parasitic, bacterial, or viral infection, either directly through receptors namely integrin receptors and pattern recognition receptors (PRRs), or through pathogen: immunoglobulin complexes *via* Fc and complement receptors. Platelets can also respond to pathogens indirectly *via* their interactions with leukocytes and the vascular endothelium at the area of local injury or inflammation. Recognition of the pathogen by platelets leads to their activation. The activated platelets through various possible mechanisms can directly kill or sequester the pathogen or orchestrate pathogen clearance by activating neutrophils and macrophages. Platelet neutrophil interaction promotes the formation of neutrophil extracellular traps (NETs) as part of the innate immune response. However, NETs can enhance platelet activation, platelet aggregation and formation of microthrombi. Intravascular platelet activation can occur as an aberrant response and if unchecked may lead to the exacerbation of inflammation, promoting the endothelial dysfunction and thrombosis. Consequently, these events can be injurious to the host and cause thrombo‐inflammation (11). The term thrombo-inflammation broadly describes a phenomenon that exhibit thrombus formation as the result of crosstalk between inflammation including immune cells and coagulation, which encompasses an array of diseases (deep vein thrombosis, stroke, atherosclerosis, and infectious diseases). The concept of thrombo‐inflammation was also used to define the role of the platelets in inflammatory response followed by cerebral ischemia‐reperfusion injury (12).

**Figure 1:**
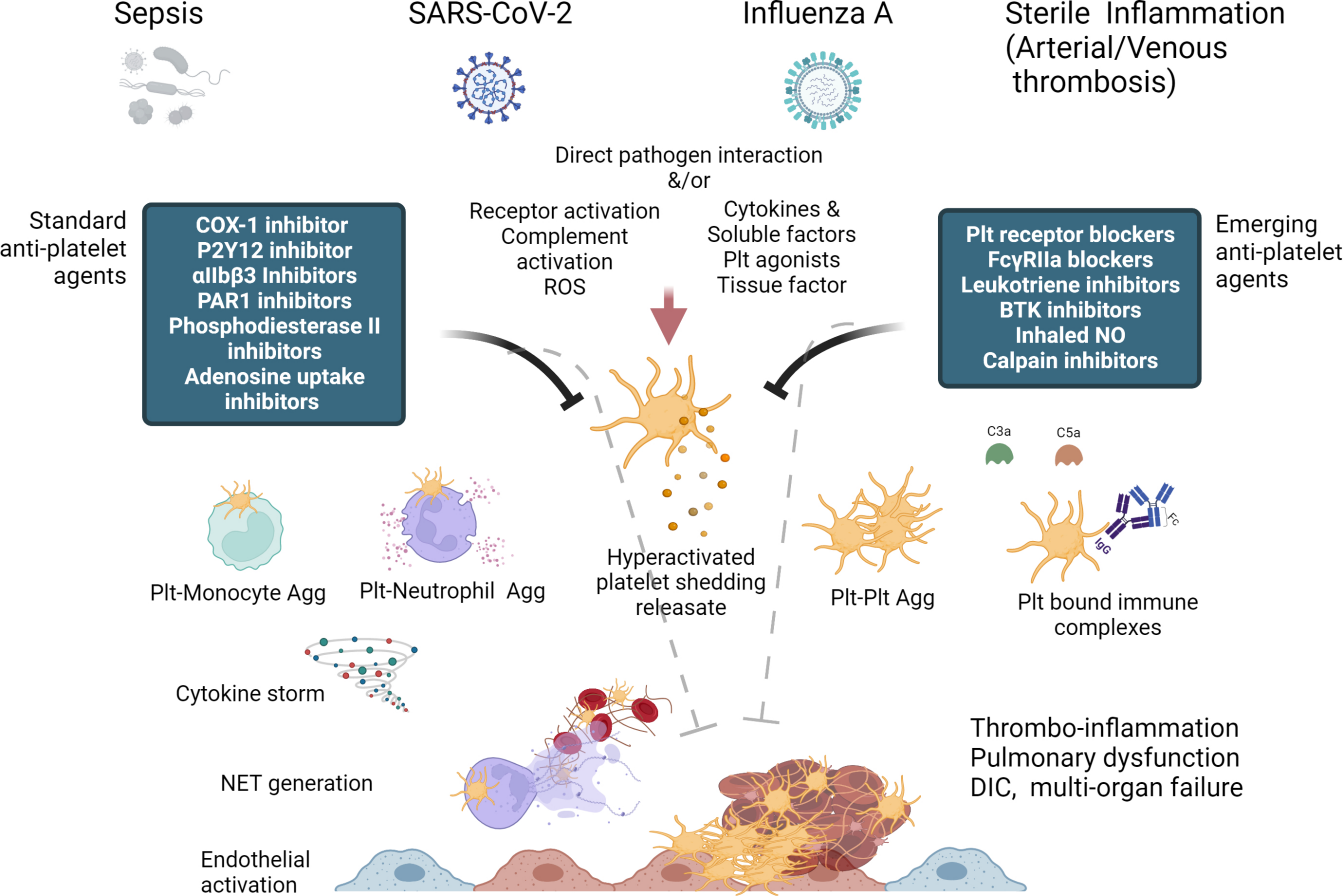
Diagrammatic summary of Platelet hyperactivation and dysfunction in thrombo-inflammation and potential of standard and emerging anti-platelet therapies, some of which can act as both anti-thrombotic and anti-inflammatory in their action. (Created with BioRender.com).

With the increase in the breadth of our knowledge regarding the complexity of the platelets and their signaling during the last decade, it is well established now that there is a crosstalk between hemostasis, thrombosis, and inflammation and they are tightly interconnected with each other. Importantly, platelets are also the key effector cells that bridge and link these three processes. Thus, this narrative review attempts to address the role of platelets in both sterile and non-sterile thrombo-inflammation with major focus on role of platelets signaling in viral infection induced thrombo-inflammation including Coronavirus disease 2019 (COVID-19). **Figure 1** summaries the theme of the present review. The last section also summaries the recent developments in deciphering the role of the main platelet receptors in thrombo-inflammation and discuss their potential as potential pharmacotherapeutic targets. As thromo-inflammation is common manifestation of both sterile (deep vein thrombosis [DVT], atherosclerosis and infectious disease [viral and bacterial infections]), it is important to study how platelets differentially regulates these processes.

The versatile functions performed by platelets can be attributed to various receptors present on them. Platelets have receptors like glycoprotein (GP) Ib-V-IX, GPVI and α2β1 that aids in adhesion to the damaged vasculature. Platelet surface receptors that respond to soluble agonists are P2Y12, thromboxane receptor and protease-activated receptor (PAR) 1 and 4 (13). Together, they belong to the G-protein coupled receptor family and mediate the second wave of platelet activation. Pathogen binding and the cellular activation of platelets is mediated by receptors like integrins, lectins and toll-like receptors (TLRs) (14, 15). Immune complexes are recognized by Fc receptor FcγRIIA present on human platelets. Infection-associated thrombocytopenia is due to an increase in platelet activation (16, 17). Activated platelets bind to leukocytes including neutrophils, monocytes, eosinophils and lymphocytes which forms heterotypic cell aggregates. Activated platelets also express P-selectin (CD62P) on their surface and released on extracellular vesicles (EVs) and harbor an array of cytokines, such as IL-1β and chemokines, such as RANTES (CCL5) (18).

## 2 Role of platelets in sterile and non-sterile inflammation

### 2.1 Sterile inflammation

#### 2.1.1 Atherosclerosis

In patients with atherosclerosis, which is a thrombo‐inflammatory disorder, the inflammatory and immune response to the endothelial dysfunction and oxidized lipids leads to formation of atherosclerotic plaques. There is accumulation of platelets and leukocytes at the site of atherosclerosis, that promotes plaque growth and progression. This eventually leads to destabilization of the underlying endothelial cell (EC) layer resulting in plaque instability/rupture and exposure of extracellular matrix (ECM) and procoagulant factors including tissue factor (TF) (19–22). Intravascular exposure of TF leads to activation of coagulation and thrombin generation. Platelets respond to ECM exposure and thrombin (primary activation) generation. In addition, platelets are also believed to be the initial regulators in the development of atherosclerotic lesions. They bind to activated ECs, leukocytes and initiate the transformation of the monocytes into macrophages. Platelets promote foam cell formation and internalize oxidized phospholipids. They also recruit progenitor cells that, depending on conditions, can differentiate into foam cells or ECs to the site of lesion. Hence, platelets not only promote progression but also regulate initiation and development of the atherosclerotic lesions (23). The platelets are recruited through GPVI‐laminin interaction, on the intact plaque promoting atheroprogression (24). Plaque rupture at the site of fissured lesions triggers the recruitment of platelets *via* GPVI‐collagen interaction. GPVI is the essential platelet collagen receptor in atherothrombosis, thus inhibition of either its extracellular domain or the downstream signaling can inhibit thrombus formation on atherosclerotic plaque. Janina et al, showed that anti-GPVI antibodies can inhibit atherosclerotic plaque-induced platelet aggregation under flow and static conditions, thus, targeting GPVI-collagen interaction along with other antiplatelet therapies in patients with spontaneous plaque rupture or intervention-associated plaque injury can be beneficial (25).

The activation of the platelets can also contribute to the pathogenesis of chronic-vascular inflammation and atherosclerosis independently of atherothrombosis. Platelets promote the uptake of oxidated low‐density lipoproteins (OxLDLs) by monocytes and macrophages, thereby increasing the monocyte recruitment and their adhesion to the atherosclerotic or inflamed endothelium. They also secrete different cytokines/chemokines contributing systemic inflammatory responses (26). Monocyte recruitment by activated platelets is mediated through interaction of P‐selectin with P-selectin glycoprotein ligand-1 (PSGL‐1) and CD40L–MAC‐1, and *via* deposition of platelet factor 4 (PF4, CXCL4) and RANTES on ECs and monocytes (27–31). Moreover, there are reports which show that PF4 downregulates the athero-protective genes in human macrophages (32). It is also reported PF4 increases OxLDL uptake by macrophages, thus exacerbating atherosclerosis (33). Studies with hyperlipidemic mice and mouse model of stroke showed that platelet PF4 forms heterodimers with RANTES, causing increased monocyte adhesion to ECs. Disruption of this interaction can be an approach to reduce atherosclerotic plaque formation (34, 35). Also, the monocyte recruitment and activation *via* platelets promote plaque instability, partly by increasing matrix metallopeptidase 9 production by monocytes (36). Factors released from activated platelets might also enhance the endothelial permeability, thus facilitating the accumulation of lipids within the vessel wall. Patients with atherosclerotic vascular disease exhibit increased number of platelet-leukocyte aggregates (PLAs). The elevated number of heterotypic PLAs in circulation is suggestive of their proinflammatory role in atherosclerosis, as it is directly corelated with higher risk of cardiovascular and cerebrovascular diseases associated with increased ECs activation (37). Due to the crucial role played by the platelets in thrombo-inflammatory events, the classical antiplatelet drugs are useful in treating thrombotic complications in arterial cerebrovascular and cardiovascular thrombosis including atherosclerosis (38, 39). P2Y12 receptor antagonists are widely used antiplatelet agents and have additional anti‐inflammatory properties that are associated with a decrease in platelet P‐selectin, circulatory PLAs, and soluble CD40L and RANTES (37). Despite having anti-thrombotic and anti‐inflammatory effects, these drugs fail to prevent the progression of already established atherosclerosis in patients. Therefore, more recently, the therapies that disrupt the PLAs have gained interest, this includes the inhibitors of P‐selectin, PSGL‐1, CD40L, and GPIb. The review by Rainger et al. summarizes the harmful and beneficial outcomes of using above mentioned inhibitors in atherosclerosis (38). Soluble CD40L is reported to plays an important role in regulating platelet-dependent thrombotic and inflammatory responses (40, 41). It mediates stimulation-induced platelet release of reactive oxygen species (ROS) through activation of Akt and p38 mitogen activated protein kinase (MAPK) signaling pathways that directs stimulation of nuclear factor kappaB and enhanced synthesis of CD40L and MCP1 (42). Increased levels of CD40L and MCP1 leads to adherence of CD40-positive cells, like platelets, to the vasculature modulating atherothrombosis (43). Along with atherosclerosis, various cardiovascular diseases (CVDs) are associated with platelet oxidative stress, thus various cardiovascular therapeutics explore altering oxidative-dependent platelet function as preventive or therapeutic measure. It has been shown that both pravastatin and aspirin inhibit expression of lectin-like oxidized LDL receptor 1 (LOX-1) on platelets by favorably affecting release of ROS from activated platelets (4). Dipyridamole at therapeutically relevant concentrations, suppresses the formation of ROS in endothelial cells and platelets thereby improving the cellular redox status (43). Polymeric flavonoids, alter platelet release of reactive oxygen intermediates with increased NO release and attenuated superoxide production, thereby leading to an immediate attenuation of release of sCD40L an important inflammatory mediator (44), The polyphenols catechin and quercetin act synergistically to reduce platelet recruitment *via* inhibition of PKC-dependent NAD(P)H oxidase activation, resulting in downregulation of NO-mediated platelet GPIIb/IIIa (45).

The use of these inhibitors is presently debatable as some of them have shown promising results in pre-clinical models, but clinical trials have been disappointing, and several studies are still on going. Also, heterogeneity and multifactorial nature of the atherosclerosis along with engagement of multiple receptors through disease progression can be held responsible for the differences observed between patients and experimental models (46).

#### 2.1.2 Deep vein thrombosis

DVT is a multifactorial disease, dictated by presence of various genetic and environmental risk factors. It is characterized by reduction of blood flow, endothelial and stromal cell activation, and coagulation dysregulation that eventually leads to thrombus formation in veins. DVT is most prevalent in the lower proximities i.e., legs under the muscular fascia of the limbs or in the central deep veins. Vascular hemostasis and thrombosis are mediated by two major mechanisms that depends on vascular damage or vessel structure (47). These two mechanisms are intrinsic and extrinsic pathways of coagulation mediated by collagen and TF, respectively. The damage to EC layer during normal hemostasis may occur which exposes the collagen from the subendothelial space. Platelets interact with the exposed collagen and von Willebrand factor (vWF) through their GPVI and GPIb/V/IX and act as mediators of primary hemostasis. The collagen exposure results in platelet adhesion and formation of the platelet monolayer at the site of vascular injury. The activated platelets result in recruitment of other circulating platelets *via* secreting various aggregatory mediators including thromboxane A2, ADP, ultra-large vWF (ulvWF) multimers. Coagulation activation leads to thrombin generation and subsequently platelet PAR activation. Also, platelet form a 3D structure through aggregating by their activated GPIIb/IIIa (α_IIb_β_3_) integrins (48). In murine DVT models, initiated by the ligation of inferior vena cava (IVC), it was reported that the platelets and leukocytes are recruited to vessel wall prior to thrombus formation (49, 50). Platelets are either recruited as small aggregates or as single cells at the site of ligation and bind to the vasculature forming heterotypic PLAs by their GPIbα receptor. The monocytes assist the DVT progression predominantly by expressing TF that triggers the extrinsic coagulation pathway The neutrophils on the other hand aid thrombus formation by interacting with platelets and releasing NETs (51). The platelet‐derived high mobility group box 1 (HMGB‐1) is proposed to be responsible for triggering NETosis in the sterile environment inside the blood vessel wall. There are studies that show that the effect of HMGB‐1 is potentiated *via* the P‐selectin–PSGL‐1 axis (52), as P-selectin deficient mice have better protection against DVT (53, 54). Platelet‐originated HMGB‐1 is known to increase the local inflammation as the result of increased increases neutrophil and monocyte sequestration at venous wall, while also promoting NETosis. The recent studies encompassing *in vivo* murine models of DVT show that complement activation also regulate the development of DVT with the components of complement displaying distinct role in thrombus formation. The activation of C3 results in platelet and fibrin deposition, whereas C5 activation results in increased TF expression on monocytes. It also promotes thrombo-inflammation by precipitating fibrin generation (55). Clinical data show increased PLAs in venous thrombosis patients (56). The high levels of platelet-neutrophil aggregates represent a risk factor for VT, as patients with DVT show correlation between platelet activation and increase in circulating platelet-neutrophil aggregates (57). Increase in platelet-monocyte aggregates were found in surgery-associated VTE (58), which correlated with increased plasma C-reactive protein levels, which itself is an important systemic proinflammatory marker (59). Anticoagulants have been keystone of contemporary therapy for thrombosis, although mechanical thrombectomy, thrombolysis and angioplasty have been also explored and used. With the emerging role of platelets in DVT, the antiplatelet drugs are also being explored for their therapeutic use to treat the disease. In particular acetylsalicylic acid (ASA), when used after initial anticoagulant treatment as a long-term secondary preventive strategy in patients with VTE, were shown to reduce risk of primary thromboembolism as well as recurrence of secondary VTE (60–62). The cardioprotective effect of ASA are mediated through irreversible inhibition of platelet cyclooxygenase 1 and blockade of the production of thromboxane A2 (TXA2). The anti-platelet effect of ASA is through reduced production of TXA2 (63).

In conclusion, initial driver of venous thrombosis is the activation of coagulation system, with significant contribution of platelets interacting with the innate immune system. These directly contribute, both systemically (in circulation) and locally (vein vasculature), to the initiation and progression of DVT and associated thrombo-inflammatory response.

### 2.2 Non-sterile inflammation driven by infection

#### 2.2.1 Sepsis

In the past decade, platelets have emerged as one of the crucial players in mediating the response to infectious disease. The ability of platelets to sense and respond to various exogenous and endogenous infectious and inflammatory signals to initiate an immune response is attributed to the wide range of complement and PRRs expressed by them including TLRs and Fc receptors (64). In response to pathogenic challenge, the activated platelets secrete their granules which contain variety of immunomodulatory and antimicrobial molecules that either aids in immune cell differentiation and activation or can directly kill pathogens. In murine models of sepsis, the platelet depletion and thrombocytopenia are associated with more severe response to disease, highlighting the protective role of platelets in sepsis (65). Early platelet transfusion has been reported to be protective in the mouse model of bacterial peritonitis (66). Platelet transfusion to reduce thrombocytopenia, was found to decrease the plasma levels of various inflammatory cytokines such as IL-6 and TNF‐α, and thus improving survival (66). The proposed underlying mechanism is macrophage polarization towards the pro‐inflammatory phenotype and subsequent increase in survival of septic mice (67). The plausible mechanism of bacterial clearance by platelets is thought to be *via* GPIb-CD11b interaction. In addition, platelet transfusion also dampens the systemic inflammation reaction by sequestering cytokines released from activation of immune cells (67). Lipopolysaccharide (LPS)-induced endotoxemia has sepsis-like features. LPS activate platelet TLR4 which enhances the release of NETs (68). NET release is mediated by P-selectins (52), release of HMGB‐1 (69) and β1‐defensins (70). Platelets also release IL‐1β-rich EVs in response to LPS-dependent TLR4 activation, which enhances ECs activation and propagation of the inflammatory response (71).

NETs and platelets are also reported to induce disseminated intravascular coagulation (DIC) which deteriorates the organ function (71). Additionally, platelets also contribute to the innate immune surveillance system of the liver as they transiently interact with the Kupffer cells that lines the walls of sinusoids in the liver through GPIbα‐vWF interaction (72). The interaction of Kupffer cells and platelets is stabilised *via* GPIIb-IIIa‐mediated platelet adhesion in presence of bacteria like *Bacillus cereus* and methicillin‐resistant *Staphylococcus aureus*. This stable adhesion leads to increased infiltration of neutrophils to the liver sinusoids and evoke host response against the pathogen (73).

Studies show that, platelets through TLR4 act as inflammatory sentinels and thus surrounds and isolate an infection, along with modulating release of proinflammatory cytokine (74). Thus, therapeutics that affect platelet-TLR signaling are being explored. Eritoran, is one such example. It competitively binds and antagonize TLR4 as mimics lipid A portion of LPS and acts as a synthetic analogue (75, 76). Therefore, it has been extensively explored as a therapeutic intervention for rampant endotoxin-mediated inflammation.

During infection, the platelets’ interactions with neutrophils or macrophages are mediated through the platelet immunoreceptor tyrosine-based activation motif (ITAM) receptors C-type lectin-like receptor 2 (CLEC‐2) and GPVI (77–79). Platelet receptors involved in PLA formation during sepsis depends on the time course of infection and the organs involved. The increase in PLAs in septic patients is inversely correlated with survival, and patients develop multiple organ failure most likely due to an increase in sequestration (80). Though during the early phases of sepsis there are elevated levels of platelet-neutrophil aggregates, but they decrease significantly with the disease progression (80). Also, the association of platelet‐monocyte aggregates with mortality depends upon age of patients, with higher mortality in older patients but not in young patients (81). Moreover, platelet depletion resulted in markedly reduced leukocyte infiltration into tissues in animal models of sepsis or ischemia reperfusion (IR) (81). Thus, in septic patients, whether targeting the platelet-immune cell interaction is beneficial or detrimental, it depends upon various other factors like disease stage and associated comorbidities.

The data from various preclinical studies support the hypothesis that the antiplatelet agents, integrin α_IIb_β_3_ antagonists or ASA, may reduce sepsis-associated mortality (82). The α_IIb_β_3_ antagonists were shown to be beneficial in animal models of sepsis as they inhibit platelet aggregation by blocking fibrinogen binding to integrin α_IIb_β_3_. In LPS-induced rat sepsis model the administration of abciximab, a Fab fragment which inhibitsα_IIb_β_3_ activation, reduced tissue edema and vascular leakage (83, 84). The use of α_IIb_β_3_ antagonists also reduced EC damage and mortality in septic mice (85). Lastly, the blockade of integrin α_IIb_β_3_ preserved red blood cell (RBC) and white blood cell (WBC) count and resulted in delayed thrombocytopenia and reduced renal damage in a baboon model of sepsis (86). Even with the success in animal models, these inhibitors have not been tested in sepsis patients. The crucial role of platelets in accelerating the septic complications is further emphasized by the study of triggering receptor expressed in myeloid cells (TREM) gene cluster. TREM-like transcript-1 (TLT-1) receptor is highly expressed in platelets and remain sequestered within platelet alpha-granules in resting platelets and is translocated to the surface after activation such as with LPS or thrombin (87, 88). Platelet TLT-1 binds to fibrinogen and facilitate platelet aggregation, and the soluble form of TLT-1 (sTLT-1) acts as a potent endogenous regulator of sepsis-associated inflammation (89). Sepsis patients exhibit elevated levels of sTLT-1, that strongly correlates with DIC score and high levels of D-dimer (89), suggestive of role of platelet-derived TLT-1 in pathology of diseases manifested as acute lung injury and acute respiratory distress syndrome (ARDS) (89).

Considering all the studies stated above, platelets play a crucial role at various stages of sepsis contributing to the thrombo-inflammatory pathophysiology and the potential use of platelet activation inhibitors as potential therapeutic approach should be explored more.

#### 2.2.2 Influenza A virus infection

Seasonal influenza infection is one of the leading causes of death by infectious disease in the United States (90). IAV is a common respiratory tract infection, that during pandemic of 1918-1919, caused 50-100 million deaths and still infects 10-20% population every year (91). In addition, IAV can cause reoccurring pandemic as seen in 2009 (92). Though adaptive immunity is required for later clearance of IAV, innate immunity plays a key role in recognition of the viral infection and initiation of immune response. It is important for viral control and clearance, but aberrant immune response can also lead to collateral damage and subsequent organ dysfunction/failure (92, 93). Severe cases of IAV infection are marked by tissue pathology and excessive inflammation and coagulation activation within the lungs (94–96). Platelets play a significant role in host defense against influenza viruses as they express various immune receptors and immune effectors upon activation by the viral invasion (97, 98). Human platelets can endocytose IAV *in vitro* and IAV viral particles have been seen within platelets in infected patients (99). In the murine model of pulmonary viral infection, a direct correlation of platelet accumulation in the lungs with the progression of disease has been reported (100, 101). Soon after IAV infection, platelets form aggregates with monocytes (102). Platelets also form PLAs with neutrophils and these PLAs transmigrate from circulation to the lung airspace of IAV infected mice (100). During viral infections, platelet-neutrophil interactions are often needed for neutrophil recruitment to site of infection, and studies demonstrates that these interactions were crucial for NET releases (101). Koupenova et al. recently showed that IAV engulfment *via* platelets causes TLR7-dependent release of complement factor C3 and subsequent activation of neutrophils and their NETosis (99). Though several recent studies validate that platelet and neutrophil recruitment to lungs drive pathogenesis of IAV, but the specific mechanisms involved have not been deciphered. The release of NETs along with their constituent neutrophil proteases, histone proteins and DNA act as primary driving forces in initiation of both thrombosis and fibrinolysis simultaneously, which form the hallmarks of associated DIC pathology (103, 104). NET generation can also act as part of antiviral immune response (105). These findings are suggestive of a vicious cycle including NET release, formation microvascular thrombosis and thrombin-induced platelet activation that together can further leads to platelet hyperactivation amplifying thrombo-inflammation and tissue damage. The recent findings by Kim et al. showed that inhibition of platelet aggregation in CD41-deficient mice or platelet-neutrophil interactions by using CD18 blocking antibodies prevents the formation of NETs and subsequent lung tissue damage in IAV infected mice (105). After IAV infection, thrombin activates platelets that promotes coagulation along with the release of inflammatory cytokines from platelets (106). The early thrombin signal generated just after the severe IAV infection is speculated to link coagulation and inflammation, further enhancing platelet-mediated thrombo-inflammatory responses (106).

Different PRRs present on platelets can bind IAV virions, further illustrating the importance of platelets in host response to influenza. These PRRs include TLR7 and TLR3 which induce prothrombotic responses by altering platelet-leukocyte interactions independent of aggregation mediated by thrombin (107). Stimulation of TLR7 on the platelet leads to surface expression of P-selectin and CD154, causing α-granule release. However, TLR3 upon stimulation, potentiates arachidonic acid-, ADP- or collagen-mediated platelet aggregation at high concentration of agonist that possibly represent late stages of infection (108, 109).

In addition, PAR1 and PAR4 are reported to be involved in progression of IAV pathogenesis (102, 110, 111). Mouse platelets lack PAR1, therefore, thrombin-mediated platelet activation occurs through PAR4 in mice (112, 113). The work by Tatsumi et al. (114) showed that global deficiency of PAR4 in mice worsens the disease outcome in the context of mild to moderate IAV infection suggestive of protective role of PAR4 during influenza infection. However, the authors were not able to separate the contribution of PAR4 on the different cell-types including platelets to IAV pathology. In line with the findings by Tatsumi et al (114),, a more recent study showed that PAR4 inhibition in wildtype mice caused more IAV-induced pathology during mild infection (105). However, during severe IAV infection PAR4 inhibition in wildtype mice seemed to mediate protection (105), which was suggested before by observations of Riteau’s group (101). The dual infection severity-dependent role of platelet PAR4 in at least IAV infection is intriguing and needs further investigation.

These studies, therefore, suggest that platelets contribute to lung injury in IAV infection likely by increasing inflammation, neutrophil recruitment/activation, and NET formation by various mechanisms. Therefore, platelet receptors can be explored for identifying reliable therapeutic targets. P2Y12 receptor antagonists are commonly used as antiplatelet agents that reduce platelet–leukocyte interactions and alter inflammatory biomarkers, associated with improved lung function in mouse models of pneumonia (80, 115). Studies showed that P2Y12 inhibition as well as ASA treatment reduced IAV pathology in mice (101, 116). Preliminary findings with P2Y12 receptor antagonists also exhibited improved lung function in humans with pneumonia (117–119).

The non-coding miR-223 regulate platelet expression of the P2Y12 receptor (120) and has been also implicated in negatively regulating TLR-NFκB signaling (121). The studies show that overexpression of miR-223 remarkably decreases pro-inflammatory cytokine production in TLR-stimulated macrophages (122, 123) and reduces neutrophilic inflammation (124). In human studies, lower plasma levels of miR-223 in patients with both stable (125) and acute (123, 126) CVD are associated with high platelet reactivity (126). In addition, ASA treatment was shown to reduce ARDS onset but did not reduce ARDS severity which may be due to the small patient numbers in those trials. These preliminary but underpowered observations underscore the need of larger multicentral clinical trials to finally test the usefulness of anti-platelet drugs in viral ARDS. The leap in knowledge for defining new pathways accelerating thrombo-inflammation warrants the study of platelet receptors belonging to the ITAM family as potential alternative therapeutic targets. For instance, the role of the ITAM receptors in IAV are not fully understood. While Fc gamma receptor (FcγRIIA) involvement in platelet activation during IAV infection was shown, the contribution of other receptors such as CLEC-2 or GPVI was unclear until recently, Boilard et al. showed Influenza virus activates platelets through FcγRIIA signaling and thrombin generation (107). Deficiency of either receptor not posing a major bleeding risk, make them potential attractive therapeutic targets in severe IAV infection (107).

Thus, the platelet-neutrophil interaction promotes acute thrombo-inflammatory responses. Also, formation of platelet-neutrophil aggregates leads to microvascular obstruction and inflammation in various thrombo-inflammatory disorders, including the acute coronary syndromes, lung injury as in infections, and ischemic stroke (119, 127, 128). The receptors involved in these interactions can be explored for their therapeutic potential. Preclinical studies have confirmed that targeting adhesion molecules on platelets like P-selectin, GPIb, α_IIb_β_3_ and on neutrophils like PSGL-1, Mac-1, inhibits the formation of neutrophil–platelet aggregates and thereby improves inflammation response and curtails microvascular dysfunction. Inclacumab, a monoclonal antibody against P-selectin in phase 1 trial has confirmed the safety and dosing as it does not increase bleeding time (128).

As the studies with new age anti-platelet drugs are still in clinical trials, it remains unclear whether emerging strategies, that primarily have cytoprotective and anti-inflammatory properties with less impact on hemostasis, will be effective. This is because previous therapeutic approaches that do not alter coagulation and tissue perfusion have not successfully improved survival or reduced organ injury. Therefore, effective, and safe therapies based on targeting platelet receptors, remains a challenge for researchers.

#### 2.2.3 SARS-corona Virus-2 infection

Initial COVID-19 patients’ autopsy studies had confirmed thrombosis occurring particularly within small vessels (platelet rich thrombotic microangiopathy) in lungs, with or without hemorrhage, which is a feature of the typical ARDS pathology of diffusely edematous lungs (129–131). As pandemic progressed, COVID-19 associated thrombo-embolic episodes included both microvascular and macrovascular thrombotic events including *in situ*-pulmonary thrombosis, and in liver and other sites (132–134), were associated with mortality. This indicated a hypercoagulable state in COVID-19 patients which involved endothelial and platelet dysfunction (135). The COVID-19 associated platelet dysfunction included reports of platelet hyperreactivity, platelet destruction and platelet-immune complex formation in patients regardless in cases of mild or severe disease progression.

## 3 Multidimensional effects of SARS-CoV-2 on platelets

The platelets can be affected by SARS-CoV-2 infection directly or indirectly. As platelets are known to have ability to interact with viruses, the reports about SARS-CoV-2 (a single stranded RNA virus) interaction with platelets were not surprising. The known receptors for SARS-CoV-2 entry in human cells include angiotensin-converting enzyme 2 (ACE-2) and transmembrane protease serine 2 (TMPRSS-2). The conflicting reports on ACE-2 expression in platelets imply that ACE-2 presence in platelets is arguable and may only be present, if at all, in very low amounts in a fraction of patients (136). TMPRSS2 is another receptor, which has also been suggested to be possible mediator for SARS-CoV-2 interaction with platelets (137). Besides ACE-2, SARS-CoV-2 spike protein can interact with integrin α_5_β_1_, the fibronectin receptor, on ECs inducing a proinflammatory phenotype (138). Moreover, α_5_β_1_ mediates SARS-CoV-2 infection in an ACE2-independent way (139). Importantly, platelets express α_5_β_1_ and a direct interaction of SARS-CoV-2 spike protein with platelet α_5_β_1_ is therefore possible. Indeed, the α_5_β_1_ binding peptide ATN-161 can block SAR-CoV-2 infection and was further showed to reduce platelet activation (140, 141). While mechanism of entry of SARS-CoV-2 in platelets may not be confirmed, its presence (Viral RNA traces, proteins, and whole virus) in patient platelets has been reported by several studies (136, 142). A remarkable study demonstrated the SARS-CoV-2 induced programmed cell death and necroptosis of platelets in which the virions were shown by transmission electron microscopy, to get internalized in platelets by being bound to EVs (120).

Besides, directly binding to viral particles, In COVID-19, platelets may also be influenced indirectly. As one of the hallmarks of COVID-19 pathology is *‘cytokine storm’* or ‘hypercytokinemia’ characterised by immune dysfunction involving systemic inflammation which spreads to multiple organs of patient and can lead to fatal multi organ failure (142). Such a systemic inflammation milieu resulting from a disproportionate immune system activation can activate platelet either through individual cytokines or by immune complexes formed in COVID-19 patients. Presence of autoantibodies in COVID-19 patients can also trigger platelet dysfunction. COVID-19 platelets have exhibited heparin-induced thrombocytopenia (HIT)-like features which are thought to be because of anti-PF4-heparin complex antibody generation (143, 144). However, such HIT-like features were also seen in patients without exposure to heparin (145), which indicates a complex mechanism behind this observation Other pathological factors such as hypoxemia have also been proposed to promote platelet hyperreactivity in COVID-19, as hypoxia has been known to induce platelet hyperactivation (146). Very recently, platelets were found partly desensitised along with presence of autophagosomes and even with visible traces of viral particles in COVID-19 patient samples (147). Interestingly, a platelet lipidome study found altered lipid composition of platelets in COVID-19 patients and linked it to platelet activation (148). Thus, platelets are affected at multiple levels in COVID-19.

## 4 Consequences of COVID-19 associated platelet dysfunction

The dysfunctional platelets in COVID-19, whether its hyperactivation of platelets or related to platelet death, consumption, or all of these, can complicate clinical management of COVID-19 patient in form of increased risk of thrombo-embolism, DIC or haemorrhage. Emerging platelet studies have provided some insights into intricacies of platelet dysfunction in COVID-19.

In context of haemostatic roles, several studies found platelets to be hyperreactive in COVID-19 in response to agonists. An altered transcriptional profile with enrichment in the pathways of antigen presentation, and mitochondrial dysfunction was observed by RNA sequencing of platelets in COVID-19 patients (142). Platelet surface markers for activation were also increased in a unique pattern in COVID-19 patients (149) along with platelet proteomic changes (150). Apart from themselves being hyperreactive, they were also reported to induce TF expression in monocytes from patients (151). Platelets’ contribution to plasma vWF, fibrinogen and FXII was also found to be higher in COVID-19 patients (152). More recently, a large-scale single cell platelet imaging by intelligent platelet morphometry, the investigators analyzed whole blood samples to identify platelet aggregates in nearly 90% of all COVID-19 patients (153). Interestingly, in this study, the platelet aggregates were also observed in some patients even after full recovery from COVID-19 symptoms, which indicate a long-lasting effect of COVID-19 on platelet physiology. As a large fraction of COVID-19 patients also show mild thrombocytopenia, platelet apoptosis was studied. The patients’ platelets showed more signs of apoptosis than healthy donors and even SARS-CoV-2 was demonstrated to directly cause apoptosis and necroptosis *ex vivo* (136, 150). COVID-19 associated platelet apoptosis was also found mediated by circulating immune complexes in patient’s sera (154). There are only limited data available from animal studies with SARS-CoV-2 or the related murine coronavirus (MHV, murine hepatitis virus) which can cause SARS-like symptoms and its effect on platelets. The recent study by Andrade et al. found that MHV infection caused early thrombocytopenia, hypercytokinemia and multiorgan failure and thereby mirroring some clinical features observed in moderate and severe cases of COVID-19 (155). Importantly, local lung and systemic changes trigged by the MHV infection could be prevented by inhibition of the TNF signaling pathway (156). Lack of TNFR1 or the use of the TNF inhibitor etanercept reduced thrombocytopenia, cytokine expression, lung injury and improved survival of MHV-infected mice. In support of the murine study, etanercept also reduced SARS-CoV-2 replication in human lung epithelial cells (156, 157).

Activated or dysfunctional platelets can release multiple cytokines including IL-1β, IL-7, IL-8, MCP-1, MIP-1α, HGF, MCP-3 (158, 159). An initial study on platelets from mild and severe disease COVID-19 patients, found platelets prone to release certain cytokines including IL-1β and CD40L, while many others including IFN-α, TNF-α and TNF-β, Eotaxin, and others were reduced. Interestingly, the same study also reported increase in EVs from COVID-19 platelets in non-severe cases but reduction in severe disease patients (160). However, thrombin induced platelet aggregation was found enhanced in both non-severe and severe disease (160). According to one report, the sCD40L was found to increase in early phase in COVID-19 patients while it decreased in the later phase of disease while sP-Selectin, the other platelet soluble marker showed opposite pattern (161). This suggests platelet response may be very dynamic as COVID-19 progresses.

In addition to cytokine release, platelet-leukocyte crosstalk has also been seen in COVID-19. The neutrophil recruitment to the pulmonary vasculature is an important event shared by ARDS and COVID-19 pathologies (162). NETs can trigger thrombo-inflammation leading to vascular thrombosis and significant mortality (163). The secondary capture’ event defined by activated platelet-neutrophil binding and rolling on endothelium, plays a key role in initiating immune-thrombosis (119). The secondary capture facilitates their transmigration to alveolar lumen and contributes to formation of edematous lungs, which can cause further platelet activation in turn. Platelets from COVID-19 patients have been reported by multiple studies to form increased PLAs which includes neutrophils or monocytes (151, 152). Platelet-Monocyte aggregates were present in higher numbers in critically ill COVID-19 patients than in those with non-severe symptoms (151). Thus, besides promoting increased prothrombotic tendency in COVID-19, platelets can also contribute to immune dysregulation in COVID-19 through soluble factors and direct cellular crosstalk with other immune cells.

## 5 Emerging therapeutic targets in COVID-19 associated platelet dysfunction

The COVID-19 patients were reported to contain increased titters of neutralizing antibodies, against SARS-CoV-2 components, the levels of which rise with severity of disease. The sera and IgG fractions from COVID-19 patients was able to induce procoagulant platelets. The study of these immune complexes revealed that aberrant glycosylation of Fc domain of these antibodies could activate platelets (164). The afucosylated IgG were higher in severe disease patients and found to activate platelets through platelet surface FcRIIA (165). In another study, the signalling mediators behind this activation was proposed to be PI3K-AKT pathway downstream of activated FcγRIIA receptor on platelet surface (166). Blocking the FcγRIIA could prevent IgG mediated platelet activation in COVID-19 platelets by preventing AKT and PI3K phosphorylation. The investigators also demonstrated that the pharmacological inhibition of AKT and PI3K phosphorylation by BAY1125976 or BYL719 could revert the induction of procoagulant platelets (166). PI3K-AKT signalling pathway has been shown earlier to mediate platelet activation by alpha-IIb mediated induction by agonists (167).

Another extensive study of FcγRIIA and complement pathway in platelets recently uncovered novel anti-platelet drug targets in COVID-19. The FcγRIIA signalling is also mediated by a protein tyrosine kinase Syk, whose inhibition by a pharmacological inhibitor drug fostamatinib could rescue FcγRIIA mediated platelet hyperactivation *ex vivo* (168). Fostamatinib is an FDA approved drug for first-line treatment for chronic ITP (169), and thus present a potentially safer therapeutic candidate against COVID-19 induced platelet dysfunction. Platelets express complement receptors C3aR (170) and C5aR (171) and complement components such as anaphylatoxins. Complement components have role in inflammatory cascade during COVID-19 infection (172, 173). A murine study with SARS-CoV indicated C3 as driver of disease. In line with this, blockade of C5a and/or C3a by neutralizing antibodies was reduce COVID-19 plasma induced platelet activation *in vitro*; the effect was more robust when each or both were combined with FcγRIIA blockade (168). As substantial complement activation is seen in autopsies studies in lung and kidney from COVID-19 patients (174, 175), the complement blockade strategy could offer a dual advantage of anti-platelet as well as immunotherapeutic against COVID-19 infection.

A related form of pathological platelet activation is also seen in small fraction of vaccinated COVID-19 patients who manifest Vaccinated Immune Thrombotic Thrombocytopenia (VITT). VITT is also seen to present features of HIT where autoantibodies against PF4 or viral spike protein can trigger platelet activation (176). The platelet immune complex formation in response to VITT antibodies respond in similar manner as that in case of HIT associated anti-PF4 antibodies (177). VITT associated platelet dysfunction also involves platelet FcγRIIA activation (178, 179). Intravenous IVIG could was able to limit the VITT (180). Platelet activation induced by plasma from VITT patients was also reverted by action of antiplatelet drugs – indomethacin and ticagrelor, and tyrosine kinase inhibitors, in the *ex vivo* settings (181).

The drug screening for in search of effective therapeutic against SARS-CoV-2 infection led to identification of Mpro inhibitors. Mpro is a cysteine protease in host cells which is also used by invading viruses including SARS-CoV-2 for its multiplication and propagation. One of the agents which inhibited Mpro was a Calpain VI inhibitor and Calpain II and XII inhibitors. One of the calpain inhibitors has been shown previously to inhibit the previous coronavirus SARS-CoV replication (182). Calpain is also important for platelets, where it acts by proteolysing structural platelet proteins and thus facilitating both early and late events of platelet activation and spreading (183). Calpain is also involved in endocytosis and apoptosis (146). Calpain inhibition has also been shown to restrict venous thrombosis in rat models (184). A recently manufactured A‐705053, small molecule calpain inhibitor can be orally administrated with enhanced pharmacokinetics (185). BLD-2660 is a new, artificial, orally active, small-molecule taken selective inhibitor of Calpain 1, 2, and 9 which is metabolically stable and permeable. It has been developed for the therapy of COVID-19 and is under trial (ClinicalTrials.gov Identifier: NCT04334460). Such a drug, if successfully tested for both anti-platelet and anti-viral functions and safety, can become one dual action drug against COVID-19 to treat both viral growth and associated platelet dysfunction.

Another drug target which can serve dual purpose of anti-platelet and anti-inflammatory actions in COVID-19 are leukotrienes. LTE4, a cysteinyl leukotriene has recently been found to induce platelet activation *in vitro* (186). The targeting of LTE4 by an approved clinical use drug – Monteleukast, has been shown to have anti-platelet activity. The mechanism was proposed to be preventing the surface expression of TF and P-selectin, reducing the formation of circulating monocyte– and granulocyte–platelet aggregates, and, finally, in completely inhibiting the release of TF positive-circulating EVs (186). The antiplatelet mechanism can also be indirect as this LTE4 antagonist reduces IL6 levels or other inflammatory cytokines which thereby may cause reduction of platelet activation. Monteleukast is in clinical use against bronchoconstriction and other inflammatory conditions which makes this a safer drug candidate for testing as anti-platelet and anti-inflammatory drug in COVID-19 (187). Among approved drugs, Nitric oxide (NO) promote vascular smooth muscle cell relaxation (vasodilation), inhibition of platelet activation, and reducing leukocyte adhesion and inflammation (188). Thus, inhaled NO may be a promising strategy for treatment of COVID-19 and an anti-platelet agent, and multiple trials of inhaled NO are in progress (189). Very recently, platelet GPIIb/IIIa receptor blockers eptifibatide or tirofiban have been reported to have reduced thrombus formation in blood from COVID-19 patients, in response to *ex vivo* shear stress (190).

Another possible antiplatelet target in COVID-19 could be platelet ITAM signaling including podoplanin-CLEC-2 axis. Podoplanin is expressed by lung epithelial cells and on tissue macrophages and could be released from the injured lung. The tyrosine kinase linked receptor CLEC-2 is expressed on platelets and has been reported play a critical role in thrombo-inflammation in mice (88). Recently, it is shown that targeting a non-receptor signaling kinase – Btk which is downstream of CLEC-2 has very effective anti-platelet effect in mice (191). In humans, Btk inhibitors - ibrutinib and dasatinib have recently been approved for use in B-cell malignancies and chronic myeloid leukemia/prostate cancer respectively (191, 192). *Post-hoc* analysis of these trials has shown that both ibrutinib and dasatinib significantly reduce venous thrombosis and that ibrutinib also reduces arterial thrombosis in B-cell lymphoma patients (193). Btk inhibitors including Ibrutinib have also been proposed to inhibit GPVI dependent platelet activation and thrombosis (194). Apart from its anti-thrombotic action, Btk inhibition has also been shown to reduce hyperinflammation based on myeloid cell cytokine release as demonstrated in mice model of brain ischemia/reperfusion injury (195). Thus, Btk inihibitors can also be potential drug candidates which can meet the need of new anti and anti-inflammatory therapies against COVID-19. **Table 1** summaries detail of emerging major pharmacological agents/antibodies (and their molecular targets) having anti-platelet/anti-thrombotic action to combat thrombo-inflammation being tested in COVID-19.

**Table 1:** Summarized detail of emerging major pharmacological agents/antibodies (and their molecular targets) having anti-platelet/anti-thrombotic action to combat thrombo-inflammation being tested in Covid-19.

S. No.	Class of Agent/Drug	Target receptor/pathway	Additional Standard Therapeutic Action	References
1.	FcRIIA signaling inhibitors (BAY1125976, BYL719 etc.	PI3K/AKT pathway	Unknown	(166)
	Fostamatinib	Tyrosine Kinase Syk	ITP treatment	(168, 169)
2.	Complement blocker/Inhibitors (Neutralizing antibodies)	C5a, C3a	Anti-inflammatory	(168)
3.	Mpro inhibitors (A‐705053, BLD-2660	Mpro, Calpain VI, II, III Calpain I, II, IX	Anti-viral (SARS-CoV-2)	(182, 185)
4.	Leukotriene inhibitor drug (Monteleukast)	LTE-4	Anti-inflammatory	(186, 187)
5.	Vasodialator (Inhaled NO)	cGMP generation	Vasodilatation, Pulmonary diseases & CVD treatment	(188, 189)
6.	Btk Inhibitors (Ibrutinib, Dasatinib etc.)	CLEC-2, ITAM signaling Btk Kinase, GPVI	B-cell malignancy, Anti-thrombotic	(193–195)
7.	Receptor Blockers (Eptifibatide, Tirofiban)	GPIIb/IIIa	Anti-thrombotic	(190)

(This is not an exclusive list).

Therefore, SARS-CoV-2 infection associated platelet dysfunction and thrombo-inflammation are tightly interlinked. The growing knowledge of platelet signaling and their interactions with immune cells has helped to understand platelet dysfunction and thrombo-inflammation in COVID-19. Understanding the complex multiple layers of COVID-19 associated effects on platelets and their response to affect hemostatic and immune events is the key towards development of novel effective and safer therapies.

## 6 Conclusion and future directions

**Figure 2:**
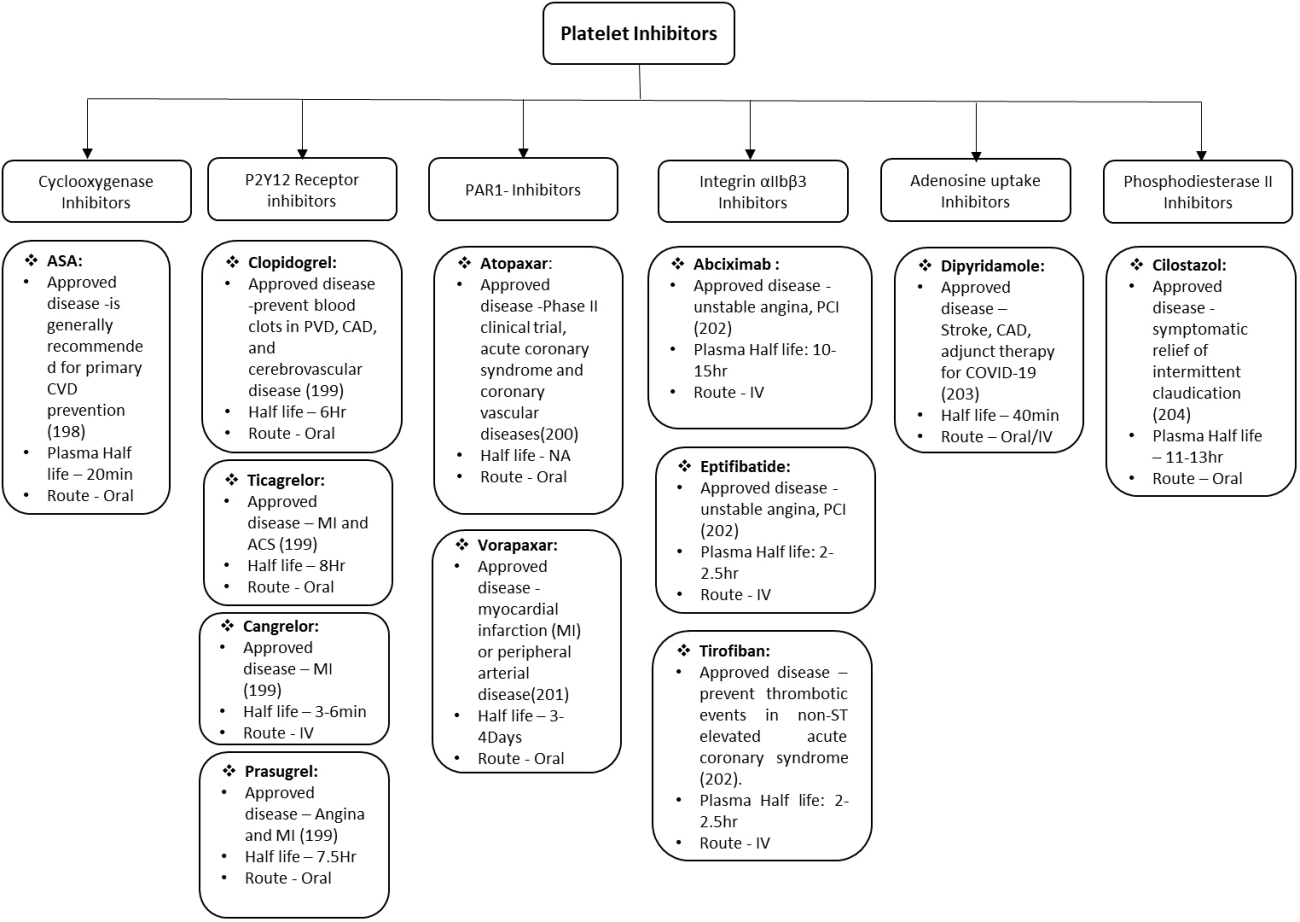
Schematic representation of Anti-platelet drugs in use and under development. ASA, acetylsalicylic acid; CVD, cardiovascular disease; PVD, pulmonary vascular disease; CAD, coronary artery disease; MI, myocardial infarction; ACS, acute coronary syndrome; IV, intra veinous; PCI percutaneous coronary intervention.

The major therapeutic challenge in the field of cardiovascular and associated diseases is to reduce the deleterious impact of microvascular thrombosis and inflammation. Even with the advent progress in therapeutics and better understanding of disease, the successful clinical management remains a formidable challenge in these disorders. This is mainly because of multifactorial and complex nature of innate immunity and hemostatic responses that directs various stages of thrombo-inflammatory process. Therefore, it is difficult to get a single “Holy Grail” that can be used to address and treat the thrombo-inflammatory complications. There are various factors that are critical for optimizing the treatment including diverse genetic background of patients, environmental cues, pathogenic heterogeneity, time of therapeutic interventions etc. Platelets are effective sentinels for sensing and responding to infection by pathogens thereby bridging hemostatic, inflammatory, and immune continuums (196, 197). The activated platelet express integrin α_IIb_β_3_, P-selectin and other receptors on the surface which play key roles in platelet activation. The activation of platelets also leads to formation of platelet aggregates and micro-thrombi, their adherence to the endothelium causing endothelial damage, increased interactions with macrophages and neutrophils, increased NETs formation, and ultimately release of cytokines. All these bridging factors added to the evolution of concept of thrombo-inflammation in various diseases. Though, studies have expanded the armamentarium of platelet functions during infectious diseases, there is lot more to be done. Specially, exploring the platelet receptors and downstream signaling for safer and effective therapeutic development. The various anti-platelet drugs depending upon the receptor they act on are summarized in **Figure 2** along with the different diseases they can prevent/manage/treat. Even with the emerging role of platelets in infectious diseases, most of clinically approved anti-platelet drugs are approved for CVDs and related complications only. In future, emerging technological innovations are likely to facilitate use of these receptors as either biomarker or therapeutic targets for infectious-inflammatory diseases as well. Developing the antiplatelet therapies which can serve dual purpose of treating unwanted hyperinflammation and thrombosis would be most effective in clinical management of thrombo-inflammatory diseases such as arterial and venous thrombosis, sepsis, influenza and COVID-19.

Though, the incredible progress in the understanding of anucleate platelet function, beyond just primary hemostasis, has been exciting journey, further discoveries will not only continue decipher role of platelets in infectious diseases but open new doors for better management and treatment of thrombo-inflammatory diseases.

## Author contributions

SS, TT and SA conceptualised the idea and wrote the manuscript. SA edited the manuscript and provided funding. All authors contributed to the article and approved the submitted version.

## Funding

The presented study was supported by grants HL142799 and HL148432 from the NHLBI to SA.

## Conflict of interest

The authors declare that the research was conducted in the absence of any commercial or financial relationships that could be construed as a potential conflict of interest.

## Publisher’s note

All claims expressed in this article are solely those of the authors and do not necessarily represent those of their affiliated organizations, or those of the publisher, the editors and the reviewers. Any product that may be evaluated in this article, or claim that may be made by its manufacturer, is not guaranteed or endorsed by the publisher.
